# Editorial Department

**Published:** 1855-01

**Authors:** 


George Hadley, M. D., Professor of Chemistry in the Buffalo Medical College, has associated himself with Mr. A. I. Mathews, the enterprising and intelligent druggist of this city, in practical chemistry. The objects of this new arrangement are such as to make it a proper subject of public comment and approval.

They intend, first, to manufacture many of the medicinal chemical products; second, to furnish analyses of soil, mineral waters, etc.; and third, to make judicial analyses whenever called upon to do so. In a word, they will be prepared to manufacture or furnish anything within the province of a working chemist.

All drugs purchased by Mr. Mathews from foreign sources, will be analyzed and warranted pure.

Prof. Hadley is widely known as a proficient both in theoretical and practical chemistry. Almost his entire life has been spent in the manipulations of the laboratory. In addition to this his character for accuracy and integrity is such as to make any analysis perfectly reliable when vouched for by him. Very many so-called agricultural chemists are in the habit of furnishing quantitative and qualitative analyses of the soils at a price below the cost of the most simple approximation to qualitative analysis alone. We can assure the public that they will not be provided with this kind of humbug by Prof. H., but if a reliable and therefore valuable analysis is required, it can be had at a fair compensation. We can speak with the same confidence of all chemicals manufactured by him for use either in medicine or the arts.

Mr. Mathews is widely known to our readers as an educated and honorable apothecary, who is doing a pure drug business, unmixed and unstained with any dealings in the patent medicine abominations. We hope to see this project successful, and trust that it may attain an importance corresponding with its merits. H.

